# Compensatory image of the stability of people with multiple sclerosis and atrial vertigo based on posturography examination

**DOI:** 10.1038/s41598-021-85983-z

**Published:** 2021-03-29

**Authors:** Oliwer Kahl, Ewelina Wierzbicka, Magdalena Dębińska, Maciej Mraz, Małgorzata Mraz

**Affiliations:** Physiotherapy Department, University School of Physical Education, al. I. J. Paderewskiego 35, 52-612 Wrocław, Poland

**Keywords:** Diseases of the nervous system, Neurological disorders

## Abstract

Pathophysiology of balance disorders due to multiple sclerosis (MS) and atrial vertigo (AV) is different. We evaluated posture stability when maintaining balance in people with MS presenting symptoms of ataxia and those with AV. We included 45 women (15 with MS; 15 with AV; 15 controls). A posturography platform was used to measure balance parameters. To characterize the image of stability and the compensation of balance disorders, the surface area of the stabilogram (SAS), vision control index (VCI) and the vision-motion control index (VMCI) were used. The stability image of people with MS and AV with eyes open (p = 0.002), with eyes closed (p = 0.080) and with visual biofeedback (p = 0.0008) differed significantly. SAS depended on visual biofeedback regardless of the occurrence of balance disorders and was the basis for determining the compensatory share of vision-motor coordination. Differences in VCI between groups were insignificant. VMCI was significantly higher in people with balance disorders than in those without, but similar in the MS and AV groups. The image of stability is different in people with MS and AV. Thanks to visual biofeedback, it becomes possible to launch effective vision-motor coordination when compensating balance disorders. VCI may become the measure of compensation for balance disorders.

## Introduction

Due to the processes of body stability, it is possible to maintain the balance and safe daily functioning of a person during all his/her activities^1^. Maintaining body balance in the surrounding space is a result of cooperation between many elements of the body, among which the vision organ, vestibular organ, deep sensation receptors and muscles dominate. It is the ability to maintain the projection of the body's centre of gravity inside the support surface, which is defined by the centre of foot pressure (COP)^2^. Disorders of postural stability create the risk of falling, which results in reduced physical activity and a decreased quality of life. People who are at risk of falling include those with multiple sclerosis (MS)^3,4^ and also those with atrial vertigo (AV)^5^.

The postural instability of people with MS is a result of existing disorders in the vision organ, vestibular organ, receptors of deep sensation and muscles^6^. Paresis of the lower limbs, abnormal muscle tension, sensory disturbance, including body position in space, and numbness in the feet are the symptoms that negatively affect postural control, both in terms of information reception and the executive mechanism^7^. Disturbed balance in patients with MS increases the risk of falls, negatively affecting their mobility and independence^3,4^.

Postural instability and balance disorders in people with AV are caused by the malfunction of the vestibular organ. Therefore, atrial vertigo is defined as an illusion of the movement of the environment (usually vortex), or of one's own body, or only head, which results from vestibular system disorders^8^. The effect of labyrinth damage and asymmetrical stimulation of the vestibular nuclei is AV, which results in balance disorders^9^.

A characteristic feature of the human posture is the vertical alignment of the body axis with respect to the support plane. Such an orientation of the body in the gravitational field causes that a person is constantly exposed to loss of balance. Due to the processes related to the active control of postural balance, the effects of instability are being compensated. This means that this control provides an optimal margin of stability that allows for the effective performance of any physical activity. This happens in the conditions of the natural environment and the physiology of the balance system, and in situations changed by damage to the systems involved in the process of regaining the balance of the body. All pathological or functional changes, impairing the functioning of the control or executive system, are reflected in changes in postural stability. Thus, postural stability control relates to the dynamic issues of maintaining or restoring body posture in the case of loss of balance. The effect of maintaining balance is the obtained safe range of human body deflection in both planes (sagittal and frontal), which can be illustrated by the swinging of the posture. The basic method of objective stability assessment is a posturographic test that records the displacement of the point of application of the resultant ground reaction force (expressed as COP) using tensometric stabilographic platforms^10,11^. The basic method of assessing stability is a posturographic examination, which records the displacement of COP. Analysis of COP movements provides a lot of information about the current state of the stability of the examined person. Based on the recorded data, it is possible to assess the variability of body deflection when maintaining balance in a standing position, and this assessment is particularly important in conditions of disturbed postural stability^2^. The assessment of motor responses and motor strategies in groups of healthy people when compared to the results of sick people can give information about the inability to use the sensory organs or the inability to select information. The size of the parameters resulting from the registration of posturographic movement strategies may also characterise the image of the compensation of balance disorders.

In order to create the proper conditions to assess the efficiency of the senses involved in postural control, posturographic examinations are performed with and without visual control, as well as with a moving visual environment and on a movable and unstable surface.

Assessment of motor responses and motor strategies adequate to the ambient stimuli in healthy people in comparison to the results of people with various diseases may give information about the inability to use the sense organs or the inability to select information. The magnitude of the parameters resulting from the registration of posturographic movement strategies may also show a picture of the compensation of balance disorders. When using this assessment to monitor treatment, changes in the pattern of recorded motor reactions and postural strategies can be expected^12,13^.

Balance disorders due to MS (ataxia) are different from those due to AV. Therefore, it is important to study the compensation of these disorders, especially in the process of body stability. It is interesting to determine the compensatory participation of the senses in the process of postural control of people with MS and AV. It should be expected that there is a different image of compensation for balance disorders in examined people. Such a comparison, as well as the indication of the compensatory participation of the senses, will give the possibility of a targeted and effective selection of rehabilitation strategies for balance disorders.

The authors proposed indices calculated using the stabilographic parameter from an objective test on a strain gauge platform. They attempt to draw the attention of a wide range of doctors and physiotherapists who deal with imbalances in their patients on a daily basis, on the way of compensating for these disorders, or the lack of compensation. One of the known and used for this purpose indices is the Romberg test, which is the quotient of the SAS with eyes closed and open. The authors propose to add another index that aggregates the SAS with eyes open and eyes closed and is the ratio of the difference of these values to their sum. As both values of these fields change during posturographic examination, it was concluded that the usual ratio of the SAS with eyes open and closed has limitations. It will not show such a significant percentage share of visual control in the compensation process of the stability of the subjects. Using this parameter, an additional quantitative index will be obtained that is very useful for assessing balance disorders and monitoring and assessing the effects of treatment. And the special significance of these indices is assigned to the possibility of assessing imbalances and ways of their compensation, which becomes useful in purposeful planning of rehabilitation. It has been observed that both clinical tests and functional tests are most often used to assess the improvement in balance and walking after participation in physiotherapy programs. The authors expect that the proposed indices of visual and visual-motor control will be used in the diagnosis of balance disorders and in monitoring the treatment. Obtaining such additional quantitative parameters does not require kinetic conditions, such as head movements, whole-body movements, limitation of the support surface, disturbance of postural reactions, or the use of eye conflict. In turn, this shortens the examination time, reduces perceived anxiety during the examination and assures safety.

The purpose of the study was to assess posture stability when maintaining balance in the standing position of people with MS presenting symptoms of ataxia and those with AV. On the basis of a COP oscillation image, the compensatory contribution of vision was assessed in relation to posture control, and the efficiency of vision-motor coordination in comparison with people without balance disorders was also evaluated.

### Hypotheses

Balance disorders in people with MS and in people with systemic AV result in a compensatory image of stability when maintaining balance in the standing position.

The image of the compensation for balance disorders of people with AV is different than that of people with MS with a domination of ataxia.

## Material and methods

### Population

The study was approved by the Senate Commission for Ethics in Scientific Research at the University School of Physical Education in Wroclaw. All subjects gave their written informed consent to participate in the study. The study involved 45 women aged 40 to 64 years with the mean of 52.8 ± 5.9 years and body mass index (BMI) of 25.7 ± 5.26 on average, who were divided into three groups:

- MS group—people with diagnosed MS and possible MS according to the McDonald diagnostic criteria for MS^14,15^ with a grade of motor impairment from 1 to 5 according to the Kurtzke Expanded Disability Status Scale and a positive result of Romberg test (ataxia). The clinical condition of the included women allowed them to walk a distance of at least 200 m without assistance and showed various degrees of impairment in daily activities. The study did not include subjects with visual impairment that hinder visual control during posturographic assessment and who did not stand alone with their eyes open and closed for at least 30 s. In total, 15 women aged 44 to 59 (mean 51.1 ± 4.4) and an average BMI of 26.6 ± 7.3 were included. Patients from this group were diagnosed with relapsing–remitting MS.
- AV group—people diagnosed with systemic AV that results from the weakening of the excitability of the peripheral vestibular organ. From each patient, details of a general medical and laryngological history were collected. All of them underwent an otolaryngological examination. In order to precisely establish the aetiology of vertigo and balance disorders and to exclude their possible central origin, the following otolaryngological tests were performed: total threshold audiometry; the standard videonystagmographic examination; and the Fitzgerald Hallpike Caloric Test. In total, 15 women aged 40 to 60 (mean 52.8 ± 6.3) and with average BMI of 25.1 ± 4.5 were included.
- Control group—people without any signs of balance disorders. In total, 15 women aged 40 to 64 years (mean 54.4 ± 6.6), average BMI 25.3 ± 3.1.

The groups were homogeneous in terms of age and BMI (p = 0.1947 and p = 0.7677 for difference, respectively).

Epidemiological data show differentiation in incidence by sex, both in people with MS and in people with AV. Depending on the source, it is estimated that vertigo and balance disorders affect 46% of men and 54% of women^16^, 48% of men and 54% of women^17^, and 31% of men and 69% of women^18^. Women are three times more likely to develop MS than men^19^. Due to the high prevalence of MS and AV in women, and to increase the homogeneity of the group, we decided to include a group of women. This choice was also dictated by age and gender differences in the image of standing posture stability, proven in many studies. This became the basis for the selection of women aged 40–64 for this study ^20^. In this analysis, duration of the disease was not taken into account as many studies reportbalance disorders both at the onset of the disease and during relapses. The study conducted by Burina et al. confirm the high prevalence of balance disorders in both people with low and high disability, assessed with the Expanded Disability Status Scale^21^.

### Methods

**Figure 1:**
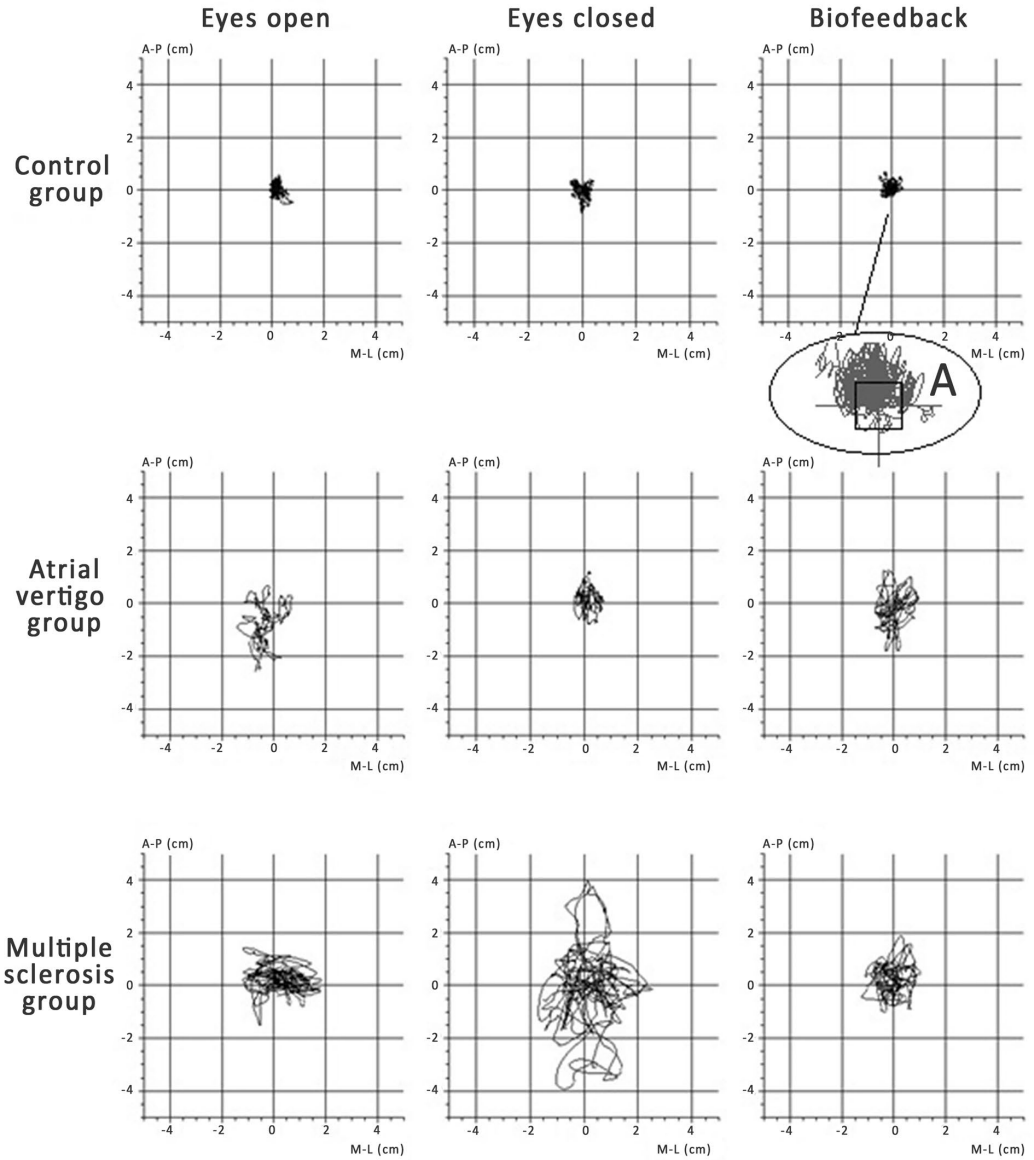
Exemplary result of posturographic examinations. *A* Conscious correction of the center of pressure position in the visual biofeedback system.

A strain gauge platform was used for testing pressure forces (Pro-Med, Legionowo, Poland). The study consisted of three 32-s measurements. Registrations of maintaining balance in the standing position were carried out with eyes open and eyes closed, and also with the use of conscious visual control—so-called visual biofeedback. The result of each measurement was a series of pairs of numbers (x, y), which defined the change in the location of the COP (the pressure of feet on the horizontally placed XY plane. Subsequent COP points are determined at consecutive times with a frequency of 32 times per second (32 Hz). These are digital samples of a continuous analogue signal. The accuracy of the analogue-to-digital conversion was 12 bits. A series of samples was saved to a disk file. All measurements were systematically calibrated, tared and cantered to the mean COP position during the initial 4 s of measurement. The resulting COP oscillation curve is called a statokinesiogram (Fig. 1). Under the actual measurement conditions, the position of the COP point is influenced by the inertia forces arising from non-uniform movements of the centre of gravity. These movements take place in different directions with varying acceleration—they are both accelerated and delayed movements.

For the analysis of the stabilogram of the examined persons, the expanded area of the stabilogram was selected. This area is calculated as the area of a polygon with irregular shapes and is the sum of the areas of triangles where one vertex is the stabilogram centre point, and the other vertices are two consecutive COP positions—points of the recorded stabilogram. Another important measure of postural control in a standing position is the size of the stabilogram envelope. It describes area and range of COP displacement (as an image of the size of the body centre of gravity swings in a standing position). The stabilogram record and the calculated parameters (posturographic measures) can be used for any analysis. Also, measurements recorded under various measurement conditions can be compared with one another. For this reason, the comparison of the size of the SASs in the posturographic examination with eyes open and closed as well as with the visual feedback gives the possibility of assessing the efficiency of the senses involved in postural control. Assessment of the share of visual control (open eyes) and visual-motor control (feedback) can inform how to compensate for balance disorders in the studied women. The vision control index (VCI) aggregates SASs with eyes open and eyes closed. It is calculated according to the following formula:

$$\mathrm{VCI}=100 \times \frac{\mathrm{SAS} \, \mathrm{with} \, \mathrm{eyes} \, \mathrm{closed } - \mathrm{ SAS} \, \mathrm{with} \, \mathrm{eyes} \, \mathrm{open}}{\mathrm{SAS} \, \mathrm{with} \, \mathrm{eyes} \, \mathrm{closed }+\mathrm{ SAS} \, \mathrm{with} \, \mathrm{eyes} \, \mathrm{open}}$$

The results were interpreted as follows:

VCI > 0—visual compensation of balance disorders, VCI < 0—a lack of visual compensation for balance disorders.

A greater value of this index results from the greater difference between the SAS without the visual control and SAS with the visual control. Theoretically, its maximum value may approach 100%. Usually, the index is positive, which means that closing the eyes increases the field of the stabilogram. The index is the greater, the smaller the field of the stabilogram with eyes open compared to the field with eyes closed. This can be interpreted as a very significant role of visual control in maintaining the balance of the body in a standing position, which suggests the role of visual control in the compensation of balance disorders. Conversely, a lower VCI value resulting from a smaller difference between the SAS with eyes closed and the SAS with eyes open may suggest a decrease in the importance of visual control in postural stability or a lack of visual compensation for balance disorders.

The vision-motion control index (VMCI) aggregates the SASs with eyes closed and visual biofeedback. It is calculated according to the following formula:

$$\mathrm{VMCI}=100 \times \frac{\mathrm{SAS} \, \mathrm{with} \, \mathrm{eyes} \, \mathrm{closed }- \mathrm{ SAS} \, \mathrm{with} \, \mathrm{biofeedback}}{\mathrm{SAS} \, \mathrm{with} \, \mathrm{eyes} \, \mathrm{closed }+\mathrm{ SAS} \, \mathrm{with} \, \mathrm{biofeedback}}$$

The results were interpreted as follows:

VMCI > 0—effective visual-motor coordination in the compensation of balance disorders, VMCI < 0—a lack of visual-motor coordination in the compensation of balance disorders.

The greater value of the VMCI index results from the greater difference between the SAS without visual control and the SAS with conscious visual control. The index is the larger, the smaller the area with the visual biofeedback, compared to the area with eyes closed. This can be interpreted as an increase in the share of visual-motor control in maintaining the balance of the body in a standing position, which suggests the role of this control in the compensation of balance disorders. Conversely, the lower VMCI value is due to the smaller difference between the SAS without visual control and the SAS with conscious visual control. This suggests a decrease in the importance of visual-motor control in postural stability or a lack of visual-motor compensation for balance disorders.

The authors of the study used a standard posturographic examination in the study protocol (examination on a hard surface). The examination on a soft surface (foam) was not possible due to the presence of ataxia in people with MS and instability as a consequence of dizziness. Those symptoms in studies subjects increased the risk of interrupting the examination or even falling^10^.

### Study conduct

The examined person stood on the posturographic platform barefoot. The position of the feet while standing was marked by lines on the platform. The examined person had to adopt a habitual position with both arms hanging freely at her sides. During the examination, the patient’s eyes were focused straight ahead. The commencement of the study was informed each time using the command “start”. Each 32-s measurement was separated by a short break, eliminating discomfort resulting from maintaining a standing position for a long time. Each patient was safeguarded by the staff standing nearby. A one-time standard examination was conducted:

- stabilographic examination with eyes open—the examined person maintained balance in a standing position, controlling the surroundings with the eyes directed towards her;
- stabilographic examination with eyes closed—the examined person maintained balance in a standing position without visual control, secured by a person standing nearby;
- stabilographic examination with visual biofeedback—on the screen placed in front of the patient, 2 m from the platform, at the level of the subject’s line of sight, a graphic object corresponding to the current COP position appeared, mapped to the screen in the form of a spot. This object performed movements on the screen that mirrored the movements of the COP. Observation of the position of the object representing the COP enabled conscious correction of the COP position. Thanks to this, allowing keeping the spot in a given position with an accuracy which was largely due to the visual-motor coordination of the examined person. The patient’s task was to observe the spot corresponding to the current COP position and to correct her posture while standing on the platform to keep this spot in the designated area (the square in the coordinate system, Fig. 1A).

Statistical calculations were made using the package of STATISTICA version 13.3 (StatSoft, Tulsa, OK, USA). The normality of the distributions of the studied variables was assessed based on the Shapiro–Wilk test. The obtained variables did not show a distribution that is consistent with a normal distribution. Descriptive statistical analysis was performed, taking into account the determination of median and standard deviations for the analysed quantitative variables. Friedman ANOVA analysis and Kendall’s compliance factor were used in order to assess whether dependent variables differ within groups, and then the Wilcoxon test was used to assess which measurements are different. In order to assess whether there are significant differences between groups, a one-way analysis of variance was used for Kruskal–Wallis ranks, which was supplemented with a post hoc test to assess differences between all pairs. To avoid the second type of error, the significance level α = 0.10 was adopted in the nonparametric tests.

### Ethics approval

The study was approved by the Senate Commission for Ethics in Scientific Research at the University School of Physical Education in Wroclaw and have therefore been performed in accordance with the ethical standards laid down in the 1964 Declaration of Helsinki and its later amendments.

### Consent to participate

All subjects gave their written informed consent to participate in the study.

## Results

### Stability image based on the size of SAS in people with balance disorders and in controls

**Figure 2:**
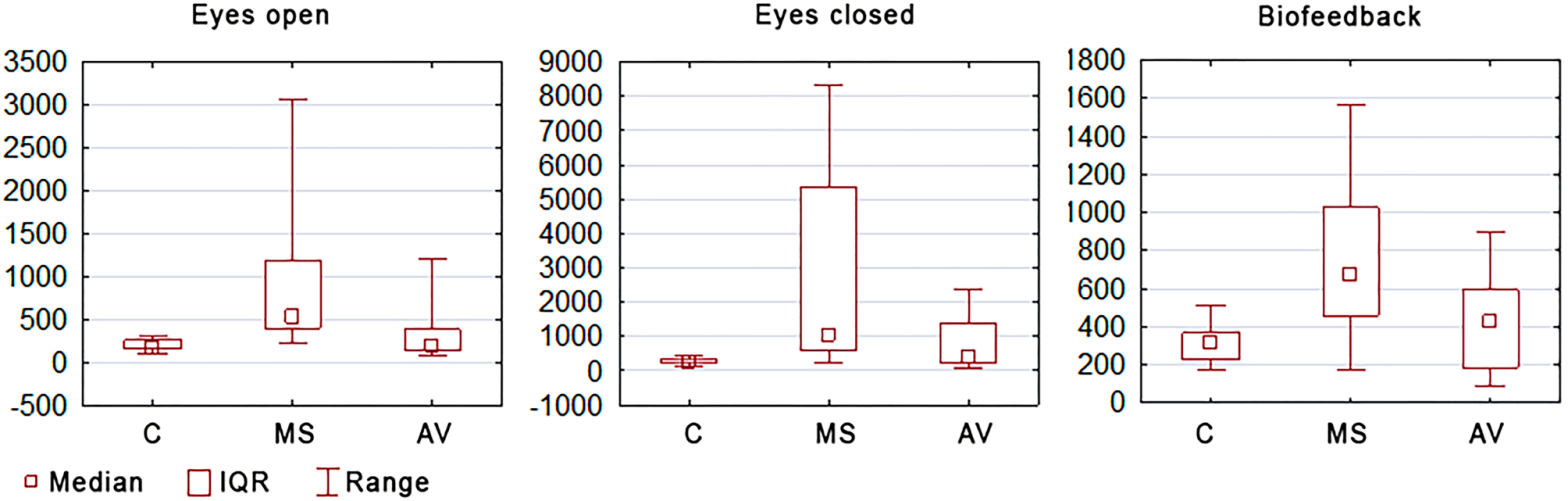
Comparison of the sizes of stabilogram surface areas between groups. *C* control group, *MS* multiple sclerosis group, *AV* atrial vertigo group.

The main effect, assessed using Kruskal–Wallis ANOVA analysis, showed significant differences in SAS in the tested groups with eyes open (p = 0.0001), with eyes closed (p = 0.0003), and also when using visual biofeedback (p = 0.0010). The post hoc analysis showed a significant variation in the size SAS when maintaining balance in the standing position with eyes open between people with MS and AV (p = 0.002), and also between people with MS and the control group (p = 0.0002). There was no difference in SAS between people with AV and the control group (p = 1.000), (Table 1, Fig. 2).

**Table 1:** Area of the stabilogram, vision control index and the vision-motion control index in all the measurements.

Group	Variable	Mean ± SD	Median (range)	Variance
Control group	1. SAS (mm^2^)	195.33 ± 64.24	172.00 (102–316)	4150.52
	2. SAS (mm^2^)	288.67 ± 92.11	277.00 (132–441)	8484.10
	3. SAS (mm^2^)	312.87 ± 106.88	310.00 (171–511)	11,424.12
	VCI (%)	18.70 ± 18.44	18.75 (− 16.56–48.48)	340.07
	VMCI (%)	− 3.72 ± 15.59	− 5.62 (− 30.97–27.27)	243.05
MS group	1. SAS (mm^2^)	894.60 ± 768.77	532.00 (221–3072)	591,002
	2. SAS (mm^2^)	2741.07 ± 2719.23	1054.00 (222–8354)	7,394,199
	3. SAS (mm^2^)	774.53 ± 378.92	673.00 (170–1569)	143,582
	VCI (%)	33.12 ± 37.12	41.60 (− 41.27–90.84)	1378
	VMCI (%)	33.85 ± 33.06	32.14 (− 20.86–81.37)	1093
AV group	1. SAS (mm^2^)	334.27 ± 355.57	184.00 (85–1209)	126,429
	2. SAS (mm^2^)	796.00 ± 711.74	411.00 (102–2379)	506,569
	3. SAS (mm^2^)	434.40 ± 245.99	423.00 (90–901)	60,509
	VCI (%)	35.50 ± 23.80	29.86 (6.60–77.47)	566
	VMCI (%)	18.31 ± 27.37	17.21 (− 52.26–54.48)	749

*SAS* surface area of the stabilogram, *AV* atrial vertigo, *MS* multiple sclerosis, *VCI* vision control index, *VMCI* vision-motion control index, *1* test with eyes open, *2* test with eyes closed, *3* test with visual biofeedback.

Similar results were obtained when comparing SAS examinations in the tests with eyes closed and with visual biofeedback. A significant difference in the size of SAS in the tests with eyes closed was shown between people with MS and AV (p = 0.080), and also between the MS group and the control group (p = 0.0001). The difference was not significant between the AV group and the control group (p = 0.206), (Table 1, Fig. 2).

Significant differences in SAS were demonstrated when maintaining balance using visual biofeedback between people with MS and AV (p = 0.0008), and also between people with MS and the control group (p = 0.039). The difference was not significant between the AV group and the control group (p = 0.746) (Table 1, Fig. 2).

An average SAS value of people with MS in the test with eyes open was equal to 532 mm^2^, while for those with AV it was 184 mm^2^. The SAS increased significantly after closing the eyes and reached an average of 1054 mm^2^ for people with MS, and 411 mm^2^ for people with AV. The use of conscious visual control in the test with visual biofeedback in both these groups resulted in a significant reduction in SAS in relation to the study with closed eyes. In a measurement using visual biofeedback, the average value of SAS was 673 mm^2^ for women with MS, while in women with VA, it was 423 mm^2^. Therefore, these values can be taken as typical regarding the image of stability of people with MS and AV.

The analysis showed that the image of stability of the MS group differs from the image of stability of the AV group, regardless of the conditions in which the tests were conducted. In each case, the median value of SAS was higher for people with MS than for people with AV (Table 1, Fig. 2).

In order to deal with the strategy of compensation of the demonstrated balance disorders, the authors first examined intra-group differences in the sizes of SAS in relation to the 3 conditions of the posturographic examination. The values of SAS were compared separately in each group. Friedman ANOVA analysis in each group showed significant differences in the size of this variable under 3 test conditions: control group (p = 0.0004), MS group (p = 0.0150), and AV group (p < 0.0001). Based on these results, the Wilcoxon test analysis was performed, and it showed significant differences in the sizes of SAS with regards to the visual control of people with balance disorders. In contrast, in people without balance disorders (control group), there was a difference in SAS between the test with eyes open and eyes closed, and no difference between the test with eyes closed and visual biofeedback (Table 2).

**Table 2:** Comparison of the sizes of the surface areas in the study with eyes open and with eyes closed.

Pair of variables	Study group	T	Z	p-value
1. SAS & 2. SAS	Control group	10.50	2.81	0.0049
1. SAS & 2. SAS	VA group	0.00	3.41	**0.0007**
1. SAS & 2. SAS	MS group	13.00	2.67	**0.0076**
2. SAS & 3. SAS	Control group	40.00	1.14	**0.2560**
2. SAS & 3. SAS	VA group	13.00	2.67	**0.0076**
2. SAS & 3. SAS	MS group	10.00	2.84	**0.0045**

*SAS* surface area of the stabilogram, *AV* atrial vertigo, *MS* multiple sclerosis, *VCI* vision control index, *VMCI* vision-motion control index, *1* test with eyes open, *2* test with eyes closed, *3* test with visual biofeedback. Bold denotes statistical significant.

The MS group had a significantly greater SAS in the test with eyes closed when compared to the test with eyes open (p = 0.0076) and to the test with conscious visual control (p = 0.0045). A similar pattern was observed in the AV group, where a significantly larger SAS was observed in the test with eyes closed when compared to the test with eyes open (p = 0.0007) and to the test with conscious visual control (p = 0.0076). However, in people without balance disorders, a significantly larger SAS only occurred when the eyes were closed when compared to those with eyes open (Table 2). Conscious visual control did not show a significant effect on SAS in the case of healthy people (p = 0.2559). The result of this analysis was the basis for determining the compensatory share of vision when controlling the posture of people with balance disorders.

### The assessment of the compensation of balance disorders of the MS and AV groups based on VCI and VMCI

**Figure 3:**
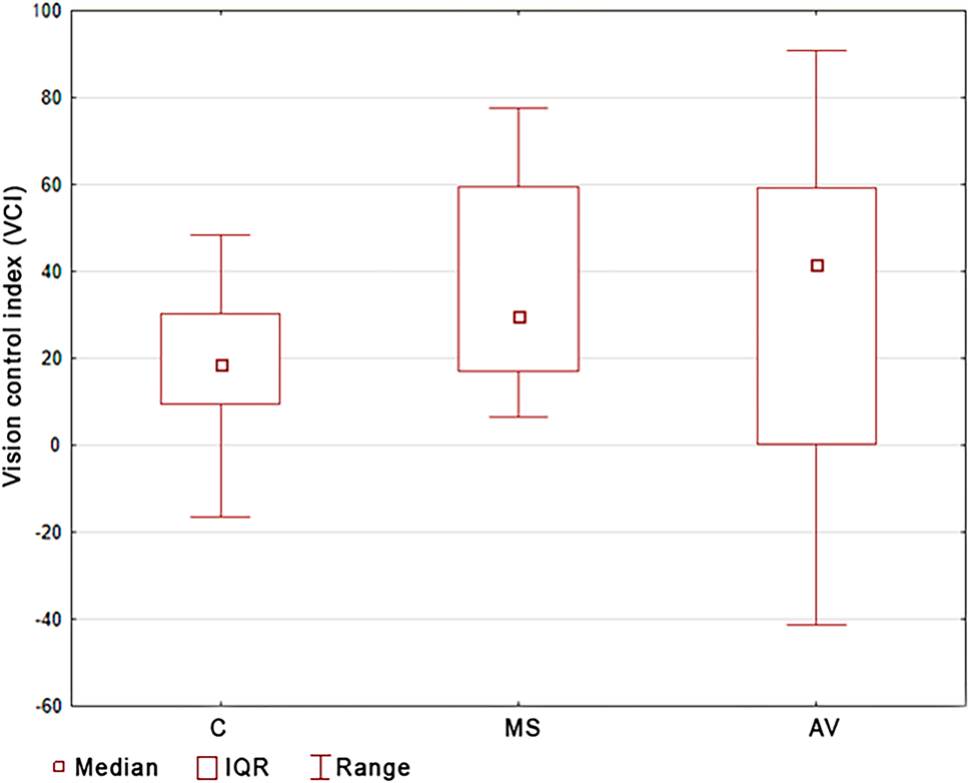
Comparison of the vision control index between groups. *C* control group, *MS* multiple sclerosis group, *AV* atrial vertigo group.

The main effect assessed using Kruskal–Wallis rank ANOVA analysis did not show statistically significant differences in VCI in the studied groups. Post hoc analysis confirmed the lack of statistically significant differences between the MS and AV group, and the control group (p = 0.1311). However, despite the lack of significant differences between the VCI groups, it is worth noting that people with MS and AV have higher values of this index than people from the control group (Table 1, Fig. 3).

**Figure 4:**
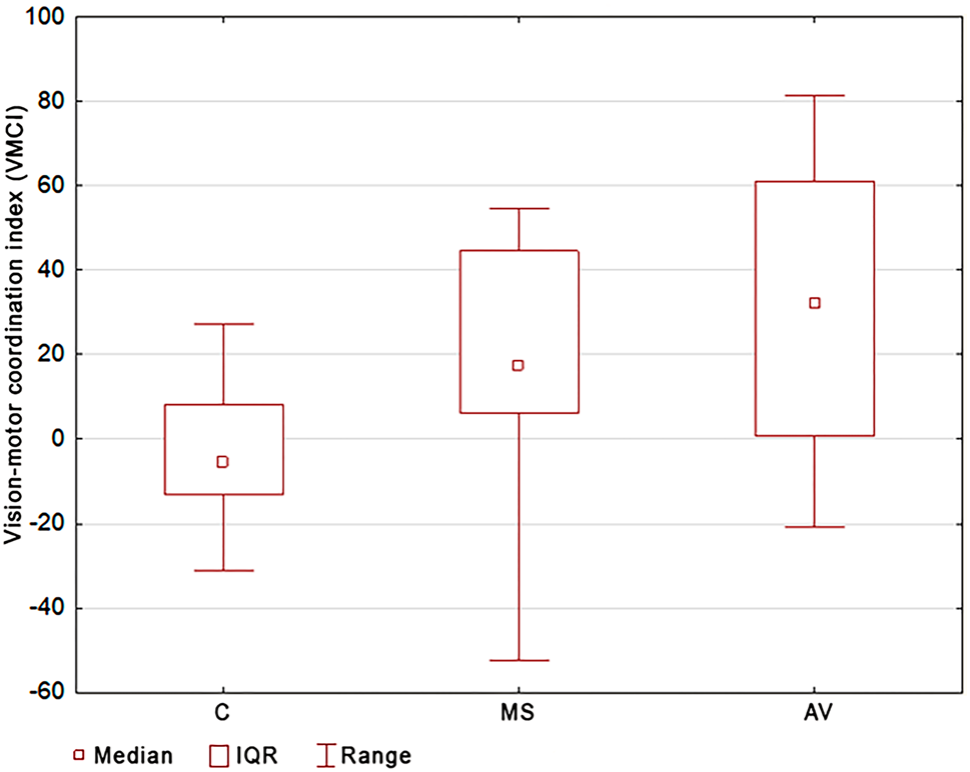
Comparison of the size of the vision-motor coordination index between the groups.

Comparison of VMCI in the examined groups showed significant differentiation (ANOVA of the Kruskal–Wallis rank, p = 0.0017). Post hoc analysis showed significant differences in the VMCI between people with AV and the control group (p = 0.037), and also between people with MS and the control group (p = 0.002). In people with balance disorders, a significantly higher rate of vision-motor coordination was observed. The median of the VMCI of the MS group was equal to 32.14%, for the AV group was equal to 17.21%, and for people without balance disorders was equal to 5.62% (Table 1, Fig. 4). There were no differences in the VMCI between the MS and AV group (p = 1.000).

## Discussion

The conducted posturographic examination evaluated the stability when maintaining balance in a standing position. This was illustrated by the surface area of the figure, which depends on the range of COP sways in all directions. The comparative analysis of the size of these areas showed that they are the largest in people with MS, which indicates the magnitude of balance disorders. Despite the subjective feelings of balance disorders in people with AV, there was no significant difference in the size of these areas when compared to the people without balance disorders. The authors also showed that the image of stability of people with MS differed from the image of stability of people with AV. In the test with eyes open and eyes closed, as well as in the test with the use of visual biofeedback, significant bigger values of this area were shown when maintaining balance in the standing position by people with MS.

An intra-group comparison of the size of the SAS was also analysed in relation to the 3 conditions of posturographic examination. It was proven that the size of these figures depended on the visual control of people with balance disorders and those without balance disorders. After closing the eyes, the size of SAS significantly increased in all subjects. This result indicates the importance of the participation of vision in the posture control of people with and without balance disorders. However, the comparison of the surface areas between the test with eyes closed and the test with visual biofeedback (conscious visual control) showed a significant difference for people with balance disorders (MS and AV), and no significant difference in people without any balance disorders. This result indicates that there is consciously activated vision-motor coordination in people with balance disorders in order to improve the stability of the standing position. The lack of a significant reduction in the surface area after starting visual feedback in the control group proves the lack of the need for conscious visual control to maintain balance in the standing position. Conscious visual control, which is an auto-correction of the symptoms of instability, is important when maintaining balance in people in whom it is disturbed. This is the basis for determining the compensatory role of vision and vision-motor coordination in people with objective and subjective symptoms of balance disorders.

The original VCI and VMCI that were used in this study showed their usefulness when assessing the compensatory participation of the senses in the process of postural control when maintaining balance in the standing position of people with MS and AV. The used indexes are an innovative method of assessing the participation of conscious vision control in postural control. They enable the quick and easy assessment of the compensation possibilities of people with balance disorders, and also the introduction of therapy that is adapted to the current state and needs of a patient. The authors showed that the participation of visual control (eyes open) when maintaining balance in the standing position is not a determinant of the compensation of imbalance in these people, and that it is rather a natural phenomenon in the situation of excluding the important sense of vision from posture control.

In contrast, the inclusion of conscious visual control as an element of visual biofeedback proved to be effective when compensating balance disorders. Thanks to visual feedback, it is possible to activate self-correction processes when maintaining balance in the standing position. The average value of the VMCI in people with MS is about 41%, which indicates such a high percentage of conscious visual control in the process of stability. In people with AV, it is about 17%. However, in the group of people without balance disorders, the average VMCI value is − 5.62%. The negative value of this index not only reveals the lack of conscious participation of vision in posture control, but also indicates destabilisation with this factor. Therefore, as demonstrated by the results of this study, it becomes possible, thanks to visual biofeedback, to launch effective vision-motor coordination when compensating balance disorders in people with such problems. The compensatory processes of postural instability in people with balance disorders (due to MA and AV) use visual-motor coordination, and the value of the VMCI may indicate the participation of conscious visual control when compensating these disorders.

The authors’ review of the literature clearly indicates the importance of the problem of balance disorders in people with MS and AV. In the available literature, the assessment of the compensation of balance disorders of people with MS and AV is limited to the analysis of the results from a standard study on a static or dynamic posturographic platform. Such a study does not examine the method of compensating these disorders, which the authors drew attention to thanks to the introduced indexes.

In 2016, Prosperini et al.^22^ conducted a study on a group of 52 MS people using a static posturographic platform. In the posturographic assessment, only one parameter was taken into account: body sway. Based on this parameter, a formula was created in order to assess the dual-task cost. The obtained results clearly indicate a higher number of body sways during a dual-task in people with MS when compared to healthy people.

Cattaneo et al.^23^ used the posturographic platform for the examination of MS people with eyes open and eyes closed. For the analysis, they used the following parameters: COP path length, the number of sways in the sagittal and lateral planes, and also velocity in the sagittal and lateral planes. They obtained the following results: MS people who are suddenly subjected to a change in environmental conditions must exert their receptor work, which may indicate the compensatory participation of the senses in posture control. The authors of this paper come to similar conclusions, showing significant differences in the VMCI between people with MS and the control group.

In the study of people with MS, Kalron et al.^4^ used static posturography, from which the following parameters were selected for further analysis: COP path length, COP surface area, and sway velocity. The tests were carried out with eyes open and closed. The results obtained by the authors pointed to disorders of the nervous system and the reduced sensitivity of receptors in people with MS, which in turn must have been compensated. The degree of compensation was not specified in the paper.

Fritz et al.^24^ examined a group of 57 MS people. The authors described the relationship between the posturographic results and gait measurements. Static and dynamic posturography was used in their research. In both cases, the amplitude of sways was analysed. The obtained results indicated the importance of posturographic examination in the assessment of long-term gait properties in people with MS.

For people with AV, standard posturographic tests, which describe the course of the COP path and which do not show how to compensate for balance disorders, are also performed. Rosiak et al.^25^ conducted a posturographic examination in a study of 50 people with AV. Tests were carried out with eyes open and eyes closed. The COP path length and COP surface area were used for the analysis. The criterion for assessing both parameters was as follows: the lower the numerical value, the better the condition of the examined person.

Zamysłowska-Szmytke et al.^26^ performed a standard posturographic examination in a study of 131 people with AV. Only one parameter was assessed: the sway speed of the centre of gravity. This work compared the result of the posturographic study with various functional scales, e.g. the Berg Balance Scale or the Dynamic Gait Index.

The study of Rosiak et al.^27^ concerning 43 people with labyrinth dysfunction was also limited to one posturographic parameter, i.e. the total length of the COP path. The effectiveness of vestibular rehabilitation with the use of both training using virtual reality and exercises on the posturographic platform was demonstrated in patients with unilateral labyrinth dysfunction. This was confirmed by objective measurements in static posturography tests, which showed an increase in postural stability with a reduction in the overall length of the COP.

The diagnosis of body balance disorders is possible thanks to posturography. Testing on the posturographic platform ensures the objectivity, reliability, high sensitivity and repeatability of measurements^4^. Due to posturography, the course of the disease can also be controlled, the progress of treatment can be monitored, and the effectiveness and purposefulness of the used rehabilitation can be assessed. The vestibular system exhibits activity in posture control mechanism when maintaining balance in the standing position on hard and soft ground, both with and without vision control. The importance of this sense increases when standing on soft ground, and even more so when the eyes are closed^28^. This mechanism is the basis for studying the compensatory image of posture stability in people with vestibular dysfunction when compared to those with MS with a predominance of ataxia.

The efficiency of the balance system is most often assessed quantitatively with the use of posturography. However, it is worth remembering that the results in the form of posturographic parameters depend on static compensation, which is not always sufficient under dynamic conditions. In posturographic diagnostics, tests with somatic input are also used, e.g. tandem stance, standing on foam^29,30^. In more innovative projects, optokinetic stimulation^31^, as well as the conscious change of position and movement of the torso and head^32,33^ are used to enhance head dizziness. Motion analysis systems that can be used in dynamic conditions are less available in clinical practice, mainly due to high costs.

For the assessment of posture control, the Modified Clinical Test of Sensory Interaction in Balance is used. It allows obtaining additional feedback from the sensory, visual, vestibular and somatosensory sensory systems^34^. However, still little is known about the validity and credibility of these tests.

Understanding the mechanisms of postural instability is fundamental to diagnosing balance disorders and treating them. Yet, the complex and diverse aetiology of balance disorders makes them challenging to diagnose and treat. Activating negative feedback aims to correct or compensate for an inadequate anticipatory reaction, counteracting the destabilisation of the pasture. For this reason, the use of multiple balance assessment tools is recommended to capture a complete picture of balance disorders and their compensations^35^. Clinical evaluation of a patient with balance disorders, supplemented by a functional test, allows to systematise the symptoms and make a preliminary clinical diagnosis. Next, based on the initial assessment, deficits that can be rehabilitated are to be identified with subsequent monitoring of the progress of rehabilitation.

The authors recognised the need to create tools that would allow the full use of the possibilities that are offered by a standard posturographic examination, and therefore they created the VCI and VMCI and demonstrated their usefulness in assessing the compensatory participation of senses in the process of postural control when maintaining balance in the standing position. The present study showed the compensatory image of the stability of people with balance disorders. Both in the MS group and the AV group, the average value of the VMCI is positive and shows a percentage share of conscious visual control, i.e. effective vision-motor coordination when compensating balance disorders.

## Limitations

In the studied clinical groups, the authors observed difficulties in the evaluation of the stability of the body in a standing position on the posturographic platform due to the existing balance disorders and the fear of falling.

In the studied clinical groups, the authors observed difficulties in the evaluation of the stability of the body in a standing position on the posturographic platform due to the existing balance disorders and the fear of falling. By choice, this evaluation was performed only once, because each repetition of the measurement had an impact on the next result. On one hand, it was better, which resulted from learning the task on the platform, and on the other hand, it was worse, due to being exposed to stress during the next attempt. Patients shared the same opinion.

For the purposes of this project, the posturographic results of a small group of people (N = 45) were analysed. The small size of the studied groups (15 subjects in each group) was due to the age and gender criteria used by the authors. This issue was explained in the description of the studied groups. Due to the impact of age and gender on the stability of a standing position, the authors created homogeneous groups in terms of age, gender and BMI to avoid a large dispersion of posturographic data. The analysis of the assessed parameters was carried out on medians values, but not average values, in order to avoid overinterpretation of the results. The authors are planning further research on larger populations.

## Conclusions

In people with MS and AV, the factors that disturb balance are different, and therefore the compensation for balance disorders is different and the values of the measures of this compensation also vary. However, in order to prove this, further research is necessary, which may allow the determination of the threshold value of these indices, and the estimation of both the risk of balance disorders and the occurrence of compensation of these disorders. This is a priority issue when planning the effective rehabilitation of people with balance disorders.

## Data Availability

My manuscript has no associated data.
